# Sleep Quality and Physical Activity as Predictors of Mental Wellbeing Variance in Older Adults during COVID-19 Lockdown: ECLB COVID-19 International Online Survey

**DOI:** 10.3390/ijerph18084329

**Published:** 2021-04-19

**Authors:** Khaled Trabelsi, Achraf Ammar, Liwa Masmoudi, Omar Boukhris, Hamdi Chtourou, Bassem Bouaziz, Michael Brach, Ellen Bentlage, Daniella How, Mona Ahmed, Patrick Mueller, Notger Mueller, Hsen Hsouna, Yousri Elghoul, Mohamed Romdhani, Omar Hammouda, Laisa Liane Paineiras-Domingos, Annemarie Braakman-Jansen, Christian Wrede, Sofia Bastoni, Carlos Soares Pernambuco, Leonardo Jose Mataruna-Dos-Santos, Morteza Taheri, Khadijeh Irandoust, Nicola L. Bragazzi, Jana Strahler, Jad Adrian Washif, Albina Andreeva, Stephen J. Bailey, Jarred Acton, Emma Mitchell, Nicholas T. Bott, Faiez Gargouri, Lotfi Chaari, Hadj Batatia, Samira C. Khoshnami, Evangelia Samara, Vasiliki Zisi, Parasanth Sankar, Waseem N. Ahmed, Gamal Mohamed Ali, Osama Abdelkarim, Mohamed Jarraya, Kais El Abed, Wassim Moalla, Nafaa Souissi, Asma Aloui, Nizar Souissi, Lisette Van Gemert-Pijnen, Bryan L. Riemann, Laurel Riemann, Jan Delhey, Jonathan Gómez-Raja, Monique Epstein, Robbert Sanderman, Sebastian Schulz, Achim Jerg, Ramzi Al-Horani, Taysir Mansi, Ismail Dergaa, Mohamed Jmail, Fernando Barbosa, Fernando Ferreira-Santos, Boštjan Šimunič, Rado Pišot, Saša Pišot, Andrea Gaggioli, Jürgen Steinacker, Piotr Zmijewski, Christian Apfelbacher, Jordan M. Glenn, Aïmen Khacharem, Cain C.T. Clark, Helmi Ben Saad, Karim Chamari, Tarak Driss, Anita Hoekelmann, on behalf of the ECLB-COVID19 Consortium

**Affiliations:** 1 High Institute of Sport and Physical Education of Sfax, University of Sfax, Sfax 3000, Tunisia; trabelsikhaled@gmail.com (K.T.); liwa.masmoudi@yahoo.fr (L.M.); omarboukhris24@yahoo.com (O.B.); h_chtourou@yahoo.fr (H.C.); hsen.hsouna92@gmail.com (H.H.); elghoulyousri@yahoo.fr (Y.E.); omarham007@yahoo.fr (O.H.); jarrayam@yahoo.fr (M.J.); kais.elabed@gmail.com (K.E.A.); wassim.moalla@gmail.com (W.M.); nafaa_souissi@hotmail.com (N.S.); 2 Research Laboratory: Education, Motricity, Sport and Health, EM2S, LR19JS01, University of Sfax, Sfax 3000, Tunisia; 3 Institute of Sport Science, Otto-Von-Guericke University, 39106 Magdeburg, Germany; anita.hoekelmann@ovgu.de; 4 Interdisciplinary Laboratory in Neurosciences, Physiology and Psychology: Physical Activity, Health and Learning (LINP2), UFR STAPS, UPL, Paris Nanterre University, 92000 Nanterre, France; tarak.driss@parisnanterre.fr; 5 Physical Activity, Sport, and Health, UR18JS01, National Observatory of Sport, Tunis 1003, Tunisia; romdhaniroma@gmail.com (M.R.); aloui.asma@gmail.com (A.A.); n_souissi@yahoo.fr (N.S.); 6 Multimedia InfoRmation Systems and Advanced Computing Laboratory (MIRACL), Higher Institute of Computer Science and Multimedia of Sfax, University of Sfax, Sfax 3000, Tunisia; bassem.bouaziz@isims.usf.tn (B.B.); faiez.gargouri@isims.usf.tn (F.G.); 7 Institute of Sport and Exercise Sciences, University of Münster, 48149 Münster, Germany; aniell.brach@uni-muenster.de (M.B.); ellen.bentlage@uni-muenster.de (E.B.); aniella.how@wwu.de (D.H.); mona.ahmad@uni-muenster.de (M.A.); 8 Research Group Neuroprotection, German Center for Neurodegenerative Diseases (DZNE), 39120 Magdeburg, Germany; atrick.mueller@dzne.de (P.M.); notger.mueller@dzne.de (N.M.); 9 Department of Neurology, Medical Faculty, Otto-Von-Guericke University, 39120 Magdeburg, Germany; 10 Programa de Pós-graduação em Ciências Médicas, Faculdade de Ciências Médicas, Universidade do Estado do Rio de Janeiro, Rio de Janeiro 20550-170, Brazil; laisanit@gmail.com; 11 Departamento de Fisioterapia, Faculdade Bezerra de Araújo, Rio de Janeiro 23052-180, Brazil; 12 Department of Psychology, Health & Technology, University of Twente, 7522 Enschede, The Netherlands; l.m.a.braakman-jansen@utwente.nl (A.B.-J.); c.wrede@utwente.nl (C.W.); sofia.bastoni2@gmail.com (S.B.); j.vangemert-pijnen@utwente.nl (L.V.G.-P.); 13 Department of Psychology, Università Cattolica del Sacro Cuore, 20123 Milano, Italy; 14 Laboratório de Fisiologia do Exercício, Estácio de Sá University, Rio de Janeiro 20261-063, Brasil; eremcarlossoares@gmail.com; 15 Department of Sport Management, Faculty of Management, Canadian University of Dubai, Dubai 117781, United Arab Emirates; mataruna@gmail.com; 16 Faculty of Social Science, Imam Khomeini International University, Qazvin 34148-96818, Iran; taheri_morteza@yahoo.com (M.T.); irandoust@soc.ikiu.ac.ir (K.I.); 17 Department of Health Sciences, Postgraduate School of Public Health, University of Genoa, 16132 Genoa, Italy; robertobragazzi@gmail.com; 18 Laboratory for Industrial and Applied Mathematics, Department of Mathematics and Statistics, York University, 4700 Keele Street, Toronto, ON M3J 1P3, Canada; 19 Department of Psychology and Sport Science, University of Gießen, 35394 Gießen, Germany; jana.strahler@psychol.uni-giessen.de; 20 Sports Performance Division, National Sports Institute of Malaysia, Kuala Lumpur 57000, Malaysia; adrianjad.isn@gmail.com; 21 Department of Sports Biomechanics, Moscow Center of Advanced Sport Technologies, 129272 Moscow, Russia; albina.andreeva@vkg.ee; 22 School of Sport, Exercise and Health Sciences, Loughborough University, Loughborough E11 3TU, UK; s.bailey2@lboro.ac.uk (S.J.B.); J.Acton@lboro.ac.uk (J.A.); E.Mitchell@lboro.ac.uk (E.M.); 23 Clinical Excellence Research Center, Department of Medicine, Stanford University School of Medicine, Stanford, CA 94305, USA; nbott@stanford.edu; 24 Computer Science Department, University of Toulouse, IRIT-INP-ENSEEIHT (UMR 5505), BP 7122 Toulouse, France; chaari.lotfi@gmail.com (L.C.); hadj.batatia@inp-toulouse.fr (H.B.); 25 UFR STAPS, UPL, Paris Nanterre University, 92000 Nanterre, France; skhoshnamie@gmail.com; 26 Onassis Cardiac Surgery Center, 17674 Athens, Greece; gelysamara@yahoo.com; 27 Department of Physical Education and Sports Sciences, University of Thessaly, 421 00 Trikala, Greece; vzisi@pe.uth.gr; 28 Consultant in Internal Medicine and Diabetes, MGM Muthoot Hospitals Pathanamthitta, Kerala 689645, India; muthootdiabcare@gmail.com; 29 Consultant Family Physician, CRAFT Hospital and Research Centre, Kodungallur, Kerala 680664, India; drwaseemahmedn@gmail.com; 30 Faculty of Physical Education, Assiut University, Assiut 71515, Egypt; mdrgamal@yahoo.com (G.M.A.); osamosama@osmail.com (O.A.); 31 Institute for Sports and Sports Science, Karlsruher Institut für Technologie, 76131 Karlsruher, Germany; 32 Department of Health Sciences and Kinesiology, Georgia Southern University, Statesboro, GA 30458, USA; briemann@georgiasouthern.edu; 33 PharmD, BCBS, PharmIAD, Inc., Savannah, GA 30458, USA; pharmiad@comcast.net; 34 Institute of Social Science, Otto-Von-Guericke University, 39106 Magdeburg, Germany; jan.delhey@ovgu.de; 35 FundeSalud, Department of Health and Social Services, Government of Extremadura, 06800 Merida, Spain; jonathan.gomez@fundesalud.es; 36 The E-Senior Association, 75020 Paris, France; monique.epstein@gmail.com; 37 Department of Health Psychology, University Medical Center Groningen, University of Groningen, 9712 Groningen, The Netherlands; r.sanderman@umcg.nl; 38 Sports- and Rehabilitation Medicine, Ulm University Hospital, Leimgrubenweg 14, 89075 Ulm, Germany; schulz.sebi@gmx.de (S.S.); achim.jerg@posteo.de (A.J.); juergen.steinacker@uniklinik-ulm.de (J.S.); 39 Department of Exercise Science, Yarmouk University, Irbid 21163, Jordan; raalhorani@yu.edu.jo; 40 Faculty of Physical Education, The University of Jordan, Amman 11942, Jordan; taiysir@hotmail.com; 41 PHCC, Primary Health Care Corporation, Doha 3050, Qatar; idergaa@icloud.com; 42 Digital Research Centre of Sfax, Sfax 3000, Tunisia; ohamed.jmaiel@redcad.org; 43 Laboratory of Neuropsychophysiology, Faculty of Psychology and Education Sciences, University of Porto, 4200-135 Porto, Portugal; fernandobarbosa@me.com (F.B.); frsantos@fpce.up.pt (F.F.-S.); 44 Institute for Kinesiology Research, Science and Research Centre Koper, Garibaldijeva 1, 6000 Koper, Slovenia; bostjan.simunic@zrs-kp.si (B.Š.); rado.pisot@zrs-kp.si (R.P.); sasa.pisot@zrs-kp.si (S.P.); 45 Department of Psychology, Catholic University of the Sacred Heart I UNICATT, 20123 Milano, Italy; andrea.gaggioli@unicatt.it; 46 Faculty of Physical Education, Jozef Pilsudski University of Physical Education in Warsaw, 00-809 Warsaw, Poland; piotr.zmijewski@insp.waw.pl; 47 Institute for Social Medicine and Health Economy, Otto-Von-Guericke University, 39106 Magdeburg, Germany; christian.apfelbacher@med.ovgu.de; 48 Exercise Science Research Center, Department of Health, Human Performance and Recreation, University of Arkansas, Fayetteville, AR 72701, USA; jordan@neurotrack.com; 49 UVHC, DeVisu, Valenciennes, LIRTES-EA 7313, Université Paris Est Créteil Val de Marne, 94000 Creteil, France; aimen.khacharem@gmail.com; 50 Centre for Intelligent Healthcare, Coventry University, Coventry CV1 5FB, UK; cain.clark@coventry.ac.uk; 51 Hôpital Farhat HACHED de Sousse, Laboratoire de Recherche “Insuffisance Cardiaque”, Université de Sousse, Sousse LR12SP09, Tunisie; helmi.bensaad@rns.tn; 52 ASPETAR, Qatar Orthopaedic and Sports Medicine Hospital, Doha 29222, Qatar; karim.chamari@aspetar.com; 53 Laboratory “Sport Performance Optimization”, (CNMSS), ISSEP Ksar-Said, Manouba University, Manouba 2010, Tunisia

**Keywords:** pandemic, home confinement, lifestyle behaviors, wellbeing, aging

## Abstract

Background. The COVID-19 lockdown could engender disruption to lifestyle behaviors, thus impairing mental wellbeing in the general population. This study investigated whether sociodemographic variables, changes in physical activity, and sleep quality from pre- to during lockdown were predictors of change in mental wellbeing in quarantined older adults. Methods. A 12-week international online survey was launched in 14 languages on 6 April 2020. Forty-one research institutions from Europe, Western-Asia, North-Africa, and the Americas, promoted the survey. The survey was presented in a differential format with questions related to responses “pre” and “during” the lockdown period. Participants responded to the Short Warwick–Edinburgh Mental Wellbeing Scale, the Pittsburgh Sleep Quality Index (PSQI) questionnaire, and the short form of the International Physical Activity Questionnaire. Results. Replies from older adults (aged >55 years, *n* = 517), mainly from Europe (50.1%), Western-Asia (6.8%), America (30%), and North-Africa (9.3%) were analyzed. The COVID-19 lockdown led to significantly decreased mental wellbeing, sleep quality, and total physical activity energy expenditure levels (all *p* < 0.001). Regression analysis showed that the change in total PSQI score and total physical activity energy expenditure (F_(2, 514)_ = 66.41 *p* < 0.001) were significant predictors of the decrease in mental wellbeing from pre- to during lockdown (*p* < 0.001, R^2^: 0.20). Conclusion. COVID-19 lockdown deleteriously affected physical activity and sleep patterns. Furthermore, change in the total PSQI score and total physical activity energy expenditure were significant predictors for the decrease in mental wellbeing.

## 1. Introduction

The Coronavirus disease 2019 (COVID-19), labelled by the World Health Organization (WHO) as a public health emergency of international concern [1], is one of the most alarming diseases in recent history [2]. As of April 1st, 2021, there have been approximately 130,085,369 laboratory confirmed cases and 2,838,054 deaths, globally (https://www.worldometers.info/coronavirus/ (accessed on 1 April 2021)). Although the virus can affect all age groups, older adults are at higher risk of suffering from negative outcomes, in addition to an increased rate of mortality [3]. Advancing or older age (in the fifth decade of life) is associated with an exponential increase in the accumulation of diverse deleterious changes in cells and tissues that are ultimately responsible for the development of chronic disease [4]. Therefore, older adults, especially those with underlying medical conditions such as arterial hypertension, cardiovascular disease, chronic obstructive pulmonary disease, and diabetes mellitus, are vulnerable to serious infections and death due to a markedly reduced immune function [5,6]. For instance, Niu et al. [6] reported that the incidences of severe infections in the age groups 50–64, 65–79, and 80 years and above were 19.8%, 43.2%, and 81.3%, respectively. In addition, the mortality rates of the aforementioned age groups were of 1.2%, 4.5%, and 18.8%, respectively [6]. To reduce the spread of the virus and to avoid the collapse of health systems, governments implemented containment strategies such as the isolation of all individuals suspected of COVID-19 and “social distancing” and “lock-downs” of varying stringency of entire populations [7]. Additionally, older adults were particularly advised to stay-at-home as much as possible to avoid contracting the virus [8].

The stringent public health measures, although effective in reducing person-to-person transmission of COVID-19 [9], have been shown to negatively impact individuals’ lifestyle behaviors (e.g., physical activity (PA) levels, sleep/wake behaviors, diet) [10,11] and their mental wellbeing [12,13,14].

Mental wellbeing is a multidimensional construct, which includes an array of dimensions, including positive emotions, engagement in meaningful activities, purpose in life, sense of accomplishment, and interpersonal relationships [15]. Mental wellbeing is an essential component of good health [16,17]; it is associated with reduced risk of morbidity [18], premature mortality [19], and functional decline [20]. It should be acknowledged that aging is associated with higher resilience (e.g., less reactivity to adverse life events), a positivity bias (e.g., more focus on good rather than bad), and successful use of coping strategies (e.g., attentional focus and appraisal), with many of these changes commencing in middle age [21,22]. In addition, the strength and vulnerability integration (SAVI) model posits that with increasing age, older adults become motivated to enhance positive wellbeing, but when a long-term stressor is encountered, it may also become more challenging to regulate sustained levels of arousal, making it difficult to return to homeostasis [23]. During the unprecedented COVID-19 pandemic, older adults have been facing additional stress due to awareness of the greater severity and fatality of COVID-19 virus in older people compared to younger groups [24,25]. Further, older individuals use digital technologies with less frequency than younger age groups, which could potentiate a more pronounced social isolation and loneliness compared to their younger counterparts [26]. In this context, lack of social connectedness and companionship is linked to increased depression [27,28] and suicidality [29], as well as to increased pro-inflammatory and decreased anti-viral immune responses [30]. These effects may further increase the susceptibility of this high-risk group of older adults to COVID-19 infection.

Mental wellbeing could be influenced by physical activity (PA) directly through enhancing mood [31] and indirectly through improving physical health [32]. However, despite the importance of PA in reducing mortality and morbidities [33], modern lifestyle behaviors encourage physical inactivity and sedentariness [34,35], which may be exacerbated in older adults due to containment strategies during COVID-19 [36,37,38]. Not surprisingly, sedentary behavior is a significant predictor of all-cause mortality, has been shown to negatively affect mood and depressive symptomatology, and is associated with cognitive decline in older adults [39].

Sleep, known by its role in strengthening immunity [40,41], could also affect individuals’ mental wellbeing [42,43]. Prevalence of sleep disorders is higher among older adults compared to younger age [44,45]. Given the stressful COVID-19 lockdowns, sleep problems could be aggravated in older adults, which in turn may impair their immune responses if contracting COVID-19 [46]. Studies examining the effects of COVID-19 lockdowns on sleep patterns in older adults are scarce. In a study conducted in China, Wang et al. [47] reported that older people were more likely to experience sleep disorders during COVID-19 lockdowns. Another study by Emerson [48] showed that sleep patterns were impacted for a little over 1/3 of a sample of older people from USA (*n* = 833), with 27% reporting more sleep than usual, and 16% reporting less sleep. In addition, older respondents (age range 60–70 years) were significantly more likely to report shorter sleep as a result of the pandemic [48]. To date, studies evaluating the effects of COVID-19 home isolation on mental wellbeing in older adults are limited and inconclusive. For instance, Knepple et al. [49] and Röhr et al. [50] reported a possible protective advantage with increased age, whilst Lopez et al. [51] suggest that some sociodemographic and health-related variables have an impact on older adults’ well-being during COVID-19 lockdowns. Finally, with recent evidence suggesting a prolongation of the pandemic after 2020 [52], a detailed exploration of possible impairment in older adults’ mental wellbeing, as well as an identification of its predictor factors during the COVID-19 pandemic are urgently needed. The WHO defines health as being not only disease-free, but rather as a state of physical, mental, spiritual, and social integration. Therefore, the importance of mental wellbeing, PA, healthy sleep, and nutrition during a pandemic and its consequences on these variables should be emphasized and explored. These findings may inform public health policies for promoting PA and sleep hygiene strategies in scenarios of public health restrictions. Therefore, this study sought to investigate, in quarantined older adults, whether sociodemographic variables and changes in PA energy expenditure and sleep quality were predictors of change in mental wellbeing, from pre- to during lockdown.

## 2. Materials and Methods

To elucidate the behavioral and lifestyle consequences of COVID-19 restrictions, an international online survey on mental health and multidimensional lifestyle behaviors during home confinement (ECLB-COVID19) was launched in April 2020. ECLB-COVID19 was opened on 1st of April 2020, tested by the project’s steering group for a period of one week and disseminated worldwide from 6th of April to 28th of June 2020 (12 weeks). Forty-one research institutions from Europe, North-Africa, Western-Asia, and the Americas promoted dissemination and administration of the survey. ECLB-COVID19 was administered in 14 languages including English, German, Arabic, French, Slovenian, Portuguese, Dutch, Spanish, Italian, Greek, Persian, Russian, Malayalam, and Indian. The survey included 64 questions on health, mental wellbeing, mood, life satisfaction, and multidimensional lifestyle behaviors (PA, diet, social participation, sleep, technology use, need of psychosocial support). All questions were presented in a differential format, to be answered directly in sequence regarding “pre” and “during” confinement conditions [10,11,12,13,53]. The study was conducted according to the Declaration of Helsinki. The protocol and the consent form were fully approved (identification code: 62/20) by the Otto von Guericke University Ethics Committee, Magdeburg, Germany.

### 2.1. Sample Size

The sample size was calculated according to a predictive equation described in Supplementary File S1. Five hundred eleven participants were needed.

### 2.2. Survey Development and Promotion

Following a structured review of the literature, the ECLB-COVID19 electronic survey was designed by a steering group of cross disciplinary academics and scientists (i.e., sport science, neuropsychology, human science and computer science) at the Otto-von-Guericke University (principal investigator), the University of Sfax, the University of Münster, and the University of Paris-Nanterre. The survey was then reviewed and edited by over 50 colleagues and experts worldwide. The survey was uploaded and shared on the Google online survey platform. A link to the electronic survey was distributed worldwide by consortium colleagues via a range of methods such as social media (LinkedIn™, Facebook™, ResearchGate™, Twitter™, WhatsApp™) shared in consortia faculties official pages and invitation via e-mails. The general public also assisted in survey dissemination through the promotion of the ECLB-COVID19 survey through their personal networks. The background and the aims of the survey were included in the introductory page, with ethics and consortium information for participants, and the option to choose one preferred language. This survey was open for all people worldwide, aged 18 years or older. People with cognitive impairment or decline were excluded. Before completing the survey, individuals voluntarily consented to anonymously participate in this study, allowing the use of their answers for research purposes [10].

Detailed information on data privacy and security and consent of participation as well as detailed description of the included questionnaires and its validation process have been previously published elsewhere [10,11,12,13,53] and were collected in Supplementary File S2. Additionally, a copy of the complete ECLB-COVID19 survey’s questionnaires has been previously published as Supplementary File (https://doi.org/10.1371/journal.pone.0240204.s001 (accessed on 1 April 2021)).

Given the large number of the assessed questionnaires, the present paper focuses on the SWEMWBS, IPAQ-SF and the PSQI questionnaires. Following, we provide short descriptions of these questionnaires, while more detailed information regarding the score calculation and the validation can be found in Supplementary File S3.

#### 2.2.1. SWEMWBS

SWEMWBS is a short version of the Warwick–Edinburgh Mental Wellbeing Scale (WEMWBS) [54]. The SWEMWBS uses seven of the WEMWBS’s 14 statements about thoughts and feelings, which relate more to functioning than feelings suggesting an ability to detect clinically meaningful change [55]. Total scores range from 7 to 35 with higher scores indicating higher positive mental wellbeing and with the cut points for SWEMWBS are (i) 17 or less for probable depression, (ii) 18–20 for possible depression, (iii) 21–27 for average mental wellbeing, and (iv) 28–35 high mental wellbeing [56].

#### 2.2.2. PSQI

The sleep quality was assessed by the PSQI [57]. The PSQI questionnaire is composed of 19 questions and has been shown to be reliable and valid in older adults [58]. PSQI scores >5 and ≤5 indicated, respectively, poor and good sleep qualities.

#### 2.2.3. IPAQ-SF

According to the official IPAQ-SF guidelines [59], data from the IPAQ-SF are summed within each of the basic three items (i.e., vigorous intensity, moderate intensity and walking) to estimate the weekly PA (MET min·week^−1^). Additionally, we added the total PA (sum of performed vigorous, moderate and walking activity) as a fourth item and sitting time as fifth item [10,11,12].

Based on the IPAQ recommendations for scoring protocol, participants of the study were classified in lowly active (<600 MET min·week^−1^), moderately active (600 MET min·week^−1^ ≤ PA < 3000 MET min·week^−1^), and highly active (≥3000 MET min·week^−1^) (http://www.ipaq.ki.se (accessed on 1 April 2020)).

### 2.3. Data Analysis

Data were reported as means (standard deviations) for continuous variables or number (percentages) for categorical variables. All statistical analyses were performed using the commercially available statistical software, SPSS Statistics version 23 (IBM, Chicago, IL, USA) and Microsoft Excel^®^ 2010 (Microsoft Corporation, Redmond, WA, USA). Using the Shapiro–Wilks W-test, normality of the data distribution was not confirmed. To examine mental wellbeing, PA, sedentary behavior, and sleep differences induced by the lockdown, comparisons among pre-, and during lockdown were carried out using Wilcoxon signed-rank tests. Cross-table Chi-square (*X*^2^) analysis was used to assess the changes compared with pre-lockdown, and the results are presented as numbers and proportions (*n*, %). Effect size (ES) for non-parametric tests was calculated using Rosenthal [60] formula: ES = Z/√*n*. ESs were interpreted as follows: small (0.10–0.30), medium (0.30–0.50), and large (≥0.50). A multiple linear regression was performed to assess the association of the change in mental wellbeing (dependent variable), with sociodemographic variables and change in sleep quality and PA. Statistical significance was set as *p* < 0.05, a priori. Changes between measures recorded before and during home confinement (delta (Δ) scores) were calculated as during confinement value minus the before confinement value. Percent changes were also calculated as follows: Δ (%) = (([During confinement value − before confinement value])/(before confinement value)) × 100.

## 3. Results

### 3.1. Data Set Selection and Sample Description

By the 28th of June 2020, 548 responses from older adults (aged > 55 years according to Petry [61], Coolidge et al. [62]; Laguna et al. [63]) were collected. Based on the age groups classification of Reynolds et al. [64], from the 548 participants, 76% were considered as young-old (56–65 years old), 19.1% were middle-old (66–75 years old), and 4.9% were classified as old-old and oldest-old adults (>75 years old). Removal of responses including data entry errors (*n* = 25) resulted in a selection of 523 participants. A screening of participants’ health status for eligibility against inclusion and exclusion criteria led to the exclusion of six participants with cognitive decline/impairment. The present study focuses on the final selected data set (i.e., 517 participants from 33 countries). Overall, 52.2% of the sample were females. Geographical breakdowns were mainly from European (50.1%), America (30%), Western-Asian (6.8%), and North-African (9.3%) countries. Age, schooling level, members sharing the same house, and health, employment and marital statuses are presented in Table 1.

### 3.2. SWEMWBS

Change in mental wellbeing total score and the distribution of responses in each item assessed through the SWEMWBS from pre- to during lockdown are presented in Table 2. The total score decreased significantly during vs. pre- lockdown. Additionally, statistically significant decreases were observed for each of the seven questions included within the survey.

**Figure 1:**
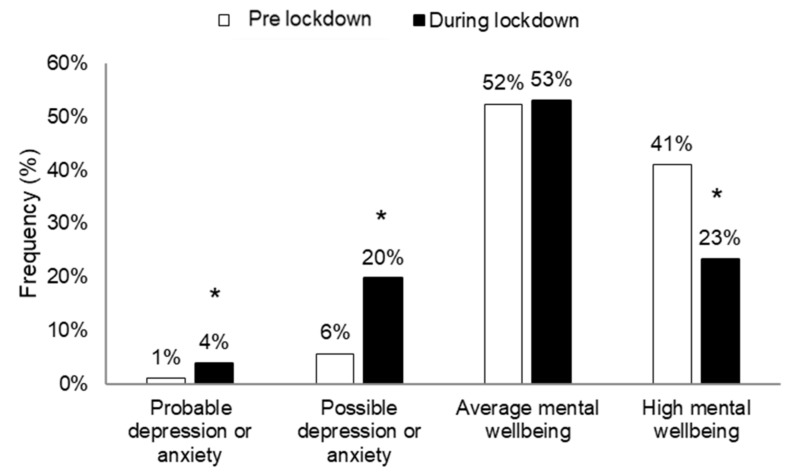
Frequencies of individuals with probable depression or anxiety, possible depression or anxiety, average mental wellbeing and high mental wellbeing pre- and during lockdown. *: significant difference between pre- and during lockdown; *p* < 0.05.

Figure 1 shows the frequencies of surveyed individuals with probable depression or anxiety, possible depression or anxiety, average mental wellbeing, and high mental wellbeing pre- and during lockdown.

The frequency of participants with high mental wellbeing decreased (*p* < 0.001), whereas the frequency of participants with probable depression or anxiety and those with possible depression or anxiety increased from pre- to during lockdown (*p* < 0.001) (X^2^_(3)_ = 74.56, *p* < 0.001, ES = 3.28).

### 3.3. PSQI

Responses to the PSQI questionnaire recorded pre- and during lockdown are presented in Table 3. Compared to pre-lockdown, sleep latency, sleep duration, subjective sleep quality score, time in bed, the score of sleep disturbances, the score of daytime dysfunctions, and the use of sleep hypnotic medication score increased, whereas sleep efficiency decreased during lockdown. The total score of PSQI increased during vs. pre- lockdown.

**Figure 2:**
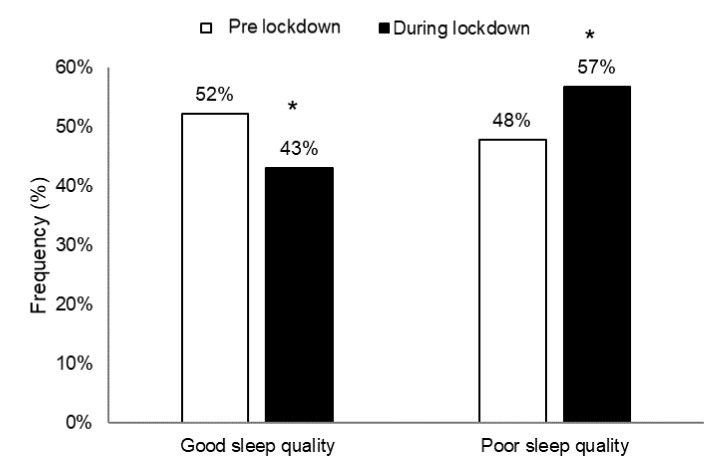
Frequency (%) of individuals experiencing a good (PSQI score ≤ 5) and bad sleep (PSQI score > 5) pre- and during lockdown. PSQI: Pittsburgh Sleep Quality Index. *: significant difference between pre- and during lockdown at *p* < 0.05.

Figure 2 shows the frequencies of surveyed individuals experiencing good and bad sleep pre- and during lockdown.

From pre- to during lockdown, the frequency of individuals experiencing a good sleep decreased (*p* < 0.05), whereas the frequency of individuals experiencing a bad sleep increased (*p* < 0.05) (X^2^_(1)_ = 8.56, *p* = 0.003, ES = 0.38).

### 3.4. IPAQ-SF

Responses to the IPAQ-SF recorded pre- and during lockdown are presented in Table 4.

Compared to pre-lockdown, the number of days/week and minutes/day of vigorous intensity, moderate intensity, and walking activities decreased during lockdown. In addition, MET values of these PA categories were significantly lower at during compared to pre-lockdown. In total, the number of days/week and minutes/day as well as the MET values of all PA recorded during lockdown significantly decreased compared to pre-lockdown. However, the amount of hours/day of sitting increased during vs. pre- lockdown.

**Figure 3:**
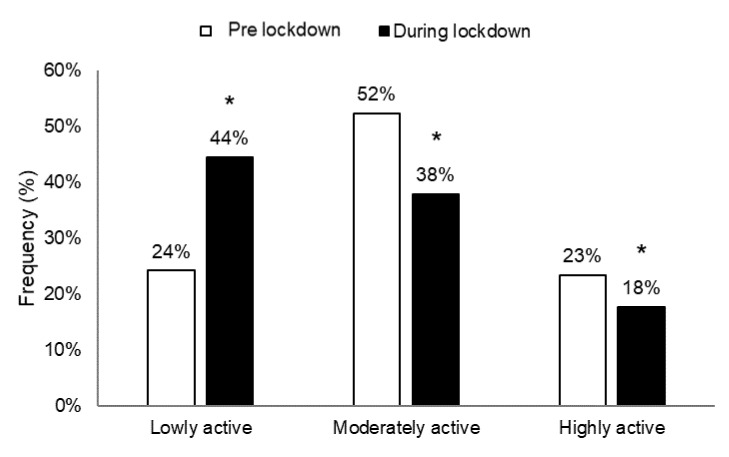
Classification of participants according to International Physical Activity Questionnaire Short Form (IPAQ-SF) scoring pre- and during lockdown. *: significant difference between pre- and during lockdown at *p* < 0.05.

The classification of respondents according to IPAQ-SF scoring pre- and during lockdown are presented in Figure 3.

From pre- to during lockdown, the frequency of high and moderate active participants decreased (*p* < 0.05), while the frequency of low active participants increased (*p* < 0.05) (*X*^2^_(2)_ = 47.35, *p* < 0.001, ES = 2.08).

### 3.5. Predictors of Mental Wellbeing Change

The results of the multiple linear regression analyses are presented in Table 5.

In the first model, all socio-demographic variables (i.e., age, sex, continent, level of education, marital status, employment status, health status, Δ house members) failed to predict ∆ mental wellbeing score. In the second model, Δ sitting was added as predictor of ∆ mental wellbeing score. However, Δ sitting failed to predict ∆ mental wellbeing score. In the third model, the addition of Δ All PA explained 9.4% the ∆ mental wellbeing score. In the fourth model, the addition of Δ PSQI explained 20.6% of ∆ mental wellbeing score. When Δ All PA and Δ PSQI were included in the final model (model 5), ∆ total PSQI score was the best single predictor of ∆ mental wellbeing score, followed by ∆ all PA. Overall, the smaller the increase in ∆ total PSQI score, the smaller the decrease in mental wellbeing levels. In addition, those who reported a smaller decrease in all PA levels, also experienced less of a decrease in mental wellbeing. Furthermore, the overall model was significant, (F_(2, 514)_ = 66.41, R^2^ = 0.20, *p* < 0.001), accounting for 20.2% (Δ PSQI: 12.3%, Δ all PA: 7.9%) of the variance in mental wellbeing score.

## 4. Discussion

The results of the present study showed an impairment in sleep quality and PA levels among older adults during COVID-19 lockdown. Additionally, a significant decrease in mental wellbeing was predicted by ∆ total PSQI score and ∆ PA levels.

### 4.1. Effects of COVID-19 Lockdown on Mental Wellbeing

A major finding of this study was the significant decrease in the levels of mental wellbeing during vs. pre-lockdown, with a mean score of SWEMWBS recorded either pre- or during COVID-19 lockdowns similar to those reported in a sample from UK (SWEMWBS score = ~23) [65]. It is worth noting that, despite the significant decrease in the levels of mental wellbeing during COVID-19 lockdown, the mean SWEMWBS scores were largely higher than 15.8 [56]; this suggests a minimal effect of COVID-19 lockdowns on mental wellbeing. The present results are in accordance with those of previous studies [49,50,66]. The minimal effect of COVID-19 lockdowns on mental wellbeing in older adults was previously explained based on the SAVI model [23]. It appears that older adults were able to regulate their own emotional reaction to a major life stressor (i.e., COVID-19 pandemic), possibly due to the fact that older adults may have faced cumulative stressors (e.g., recession, war, epidemics) and have more personal resources to deal with stressors than younger counterparts [23]. Additionally, older adults tend to apply accommodative strategies to cope with new stressful situations [67], thus reducing the perception of COVID-19 restrictions.

### 4.2. Effects of COVID-19 Lockdown on PA

All PA intensity levels (i.e., walking, moderate, vigorous) decreased significantly during COVID-19 lockdown; a finding that was previously reported in older people during the COVID-19 pandemic [36,38,68]. This marked decrease could be explained by the restriction imposed by the lockdowns and causing the closure of gymnasiums and sports halls, as well as the decrease of recreational or incidental daily PA (e.g., walking, bicycling) [14,69], and governmental guidance on restricting face-to-face contact.

It is worth noting that the percentage of lowly active individuals increased during COVID-19 lockdowns, which could be explained by the drastic change in everyday schedules and habits. For example, people staying at home during lockdowns spent much more time engaged in low-intensity activities, such as housework (e.g., cooking, washing dishes, gardening) [11] vs. outside of lockdowns.

According to the WHO [70], older adults are advised to participate in 150 min/week of moderate-intensity, or 75 min/week of vigorous-intensity, or an equivalent combination of both, for health enhancement and prevention of non-communicable diseases. However, the current findings indicate that participants were far from reaching the WHO recommendations, both pre- and during lockdown. Clearly, a more concerted effort on PA promotion in older people is urgently needed.

In the present study, daily sitting time increased significantly by two hours per day during COVID-19 lockdowns (large ES = 0.740), confirming previous results [10,11,12,36,71]. Additionally, the reported mean values in the current study are of concern as the daily older participants’ sitting time during the COVID-19 lockdown resides in the threshold area (i.e., 6–8 h), which may lead to increased risks of developing diseases and ever higher mortality [72].

### 4.3. Effects of COVID-19 Lockdown on Sleep Patterns

Consistent with the results of a previous study conducted in the general population [11], global PSQI scores increased significantly during vs. pre-COVID-19 lockdowns. In addition, the PSQI scores recorded during lockdowns were higher than the cut-off for poor sleep quality, suggesting that quarantined older people suffered from poor overall sleep quality. Moreover, the percentage of participants reporting bad sleep quality was higher during COVID-19 lockdown vs. pre-lockdown, confirming impairments in sleep quality.

Some components of the PSQI questionnaire increased significantly during vs. pre-lockdown. Sleep duration increased significantly during lockdown, a finding that was previously reported by Trabelsi et al. [11] and Lee et al. [5]. Moreover, the reported mean sleep durations pre- and during lockdowns were below the recommended level of sleep duration for older adults (i.e., 7–8 h) [73]. Previous studies reported that inadequate sleep duration was associated with several adverse health outcomes such as obesity, cardiovascular disease, cancer, type 2 diabetes mellitus, cognitive decline, as well as total and cause-specific mortality [74,75,76,77,78].

Sleep latency, another component of the PSQI questionnaire, increased significantly during vs. pre-lockdown. It should be acknowledged that the reported mean sleep latency recorded during COVID-19 lockdowns exceeded 20 min, which is indicative of sleep problems in older people [79]. The potential pre-sleep thoughts, particularly about the ease of transmission of COVID-19 and its potential mortality in older people [80], leading to anxiety and stress [81], could explain, in part, previous findings. Other factors, such as unhealthy diet behaviors, less daylight exposition, conflicting messages from authorities, financial security, and job continuity issues could also lead to difficulties in commencing and sustaining sleep during lockdowns. As a consequence, to assist falling asleep during COVID-19 lockdown, older people increased their intake of sleep-facilitating medication, as shown in the present findings.

We also found that sleep disturbances increased significantly during vs. pre-lockdowns, which could be explained by the COVID-19 situation and its associated stresses. Consequently, higher daytime dysfunctions were reported during COVID-19 lockdowns, potentially inducing more frustration and negative emotions [82].

Sleep efficiency, defined as the ratio of total sleep time to time in bed [83], decreased significantly during COVID-19 lockdowns; however, the average reported values were higher than the cut-off of 80% [83], potentially indicating protection against mortality risks in older people [84].

### 4.4. Predictors of Self-Reported Change in Mental Wellbeing

The significant decrease in mental wellbeing, although not indicating poor levels, should not be neglected; it should be taken into consideration as a harbinger of potentially greater issues given the signs of prolongation of the stressful COVID-19 pandemic. To better understand the reasons for mental wellbeing declination during COVID-19 restrictions, the associations between sociodemographic characteristics, Δ PA levels, Δ sleep quality and the magnitude of the COVID-19 lockdown effect on mental wellbeing were investigated. The results of the multiple linear regression analysis revealed Δ PA was the best single predictor of Δ mental wellbeing. The present findings showed a marked reduction in PA levels, which could possibly lead to a decrease in the release of endorphins, modulated by physical exercise, and known by their beneficial effect on mood by reducing stress and generating a feeling of euphoria [85]. Additionally, it is well recognized that PA is implicated in the modulation of circulating neurotrophins [86]. Moreover, the brain-derived neurotrophic factor, the most abundant neurotrophin, could reduce both anxiety and depressive disorders [87]; therefore, it is not surprising in the present study to find (i) a significant increase in the percentage of older people with probable and possible depression or anxiety, and (ii) a significant decrease in the percentage of older people with high mental wellbeing during lockdown.

The present results showed that Δ global PSQI score was also a significant predictor of Δ mental wellbeing. It was recently reported that reduced or disrupted sleep is a risk factor for depression and anxiety, leading to impaired wellbeing [88,89]. Additionally, sleep quality is considered as an important predictor of wellbeing in seniors [90]. Nevertheless, the relationship between psychological disorders (e.g., depression) and sleep disturbance in older adults has been hypothesized to be bidirectional, with depression increasing the risk of poor sleep and poor sleep predicting depression [91]. Future research on this topic is warranted to better elucidate the veracity of this relationship.

### 4.5. Strengths and Limitations

The main strength of this study is the use of a multicenter anonymous cross-disciplinary online survey including a number of validated questionnaires, recently recommended as an exciting and flexible qualitative research tool [92]. Furthermore, the suitably powered sample size and the rapid collection of data during the restrictions are additional strengths, both in terms of functionality and practicality. However, despite these strengths, some limitations must be considered in the interpretation of our results. Firstly, half of the participants were from European countries, which generally have smaller populations compared with India and Malaysia. While these two countries whose populations are fairly large, were not well represented. This could be related to the low use of digital technologies, particularly in India [93]. Future studies assessing the effects of COVID-19 lockdown on mental wellbeing and lifestyle behaviors in Indian and Malaysian older adults are warranted. Secondly, data collection based on online survey may lead to an underrepresentation old-old adults, possibly due to their often-limited experience and use of digital technologies. Additionally, the online advertised survey could have resulted in volunteer bias. It is possible that older adults interested in lifestyle behaviors and/or mental wellbeing during COVID-19 lockdowns could be more prone to participate and to perceive differences between pre- and during COVID-19 lockdowns. Thirdly, PA levels measurements were based on subjective descriptions rather than objective assessment, which could contribute to an overestimation of the self-reported PA levels [94]. Though, Tran et al. [95] showed that IPAQ-SF is an acceptable tool to assess PA in older adults. Fourthly, daytime napping, common among older adults [96], is unfortunately not assessed by the PSQI questionnaire. Finally, the present findings concern older adults surveyed during the initial moments of COVID-19 pandemic and do not take into account the long-term effects of the pandemic on the wellbeing of the participants. Addressing these shortcomings in future studies, using objective measurement tools (i.e., based on accelerometry), in addition to validated subjective tools (as we did), and assessing daily naps, is warranted, and may yield unseen insight into the lockdown phenomena.

## 5. Conclusions

COVID-19-related lockdown significantly and deleteriously altered sleep quality and PA levels in older adults. Sleep quality and total physical activity energy expenditure were significant predicators of the decrease in mental wellbeing from pre- to during lockdown. The public policies put in place must consider these factors as levers for improving the well-being of the population in order to effectively combat the spread of COVID-19. However, given the widespread indication of a prolonged COVID-19 pandemic, future studies investigating the long-term effects on mental wellbeing in older people are warranted. Importantly, Information and Communication Technology (ICT) based solutions (e.g., smart watch, sensors/accelerometer, apps, recommender system, virtual coach) can provide self-monitoring and home-based coaching features for older-adults during lockdowns, thus, helping then to adhere to an Active Healthy and Confinement Lifestyle (AHCL) and reduce psychosocial strain in this vulnerable population [97].

## Figures and Tables

**Table 1:** Demographic characteristics of the participants (*n* = 517).

Variables	*n*	(%)
**Age (years)**		
56–60	255	(49.3%)
61–65	138	(26.7%)
66–70	76	(14.7%)
71–75	23	(4.4%)
76–80	18	(3.5%)
>80	7	(1.4%)
**Sex**		
Female	270	(52.2%)
Male	247	(47.8%)
**Continent**		
Europe (16 countries)	259	(50.1%)
America (5 countries)	155	(30%)
North-Africa (3 countries)	48	(9.3%)
Western-Asia (4 countries)	35	(6.8%)
Others (5 countries)	20	(3.9%)
**Level of Education**		
Master/doctorate degree	256	(49.5%)
Bachelor’s degree	138	(26.7%)
High school graduate, diploma, professional degree or the equivalent	114	(22.1%)
No schooling completed	9	(1.7%)
**Marital Status**		
Single	52	(10.1%)
Married/Living as couple	376	(72.7%)
Widowed/Divorced/Separated	89	(17.2%)
**Employment Status**		
Employed for wages	239	(46.2%)
Self-employed	60	(11.6%)
Out of work/Unemployed	16	(3.1%)
Student	2	(0.4%)
Retired	169	(32.7%)
Unable to work	8	(1.5%)
Problem/unemployment caused by COVID-19	11	(2.1%)
Other	12	(2.3%)
**Health Status**		
Healthy	349	(67.5%)
With risk factors for cardiovascular disease	150	(29%)
With cardiovascular disease	18	(3.5%)
**Members Sharing the Same House**		
0 (live alone)	85	(16.4%)
1	241	(46.6%)
2	107	(20.7%)
3	55	(10.6%)
>3	29	(5.6%)

**Table 2:** Distribution of responses (%) in each item and total score of the mental wellbeing questionnaire.

Parameters	Means ± SD		Δ (Δ%)	T (Wilcoxon)	Z	*p*-Value	ES
	Pre-Lockdown	During Lockdown					
I’ve been feeling optimistic about the future	4.01 ± 0.83	3.47 ± 1.01	−0.54 (−13.5%)	1634.0	12.28	<0.001	0.78
I’ve been feeling useful	4.12 ± 0.77	3.74 ± 1	−0.38 (−9.2%)	1885.0	9.42	<0.001	0.69
I’ve been feeling relaxed	3.7 ± 0.87	3.27 ± 1	−0.43 (−11.6%)	7849.0	8.60	<0.001	0.52
I’ve been dealing with problems well	4.02 ± 0.69	3.78 ± 0.79	−0.25 (−6.1%)	1655.0	7.65	<0.001	0.62
I’ve been thinking clearly	4.2±0.67	3.93±0.83	−0.27 (−6.5%)	1398.5	8.06	<0.001	0.66
I’ve been feeling close to other people	4.11±0.76	3.6±1.04	−0.51 (−12.4%)	2977.0	10.57	<0.001	0.69
I’ve been able to make up my own mind about things	4.37±0.69	4.12±0.85	−0.25 (−5.7%)	918.5	7.82	<0.001	0.68
Total metric score	28.54±3.83	25.91±4.66	−2.63 (−9.2%)	6942.0	14.30	<0.001	0.72

SD: standard deviation; Δ%: % change from pre- to during lockdown; ES: effect size.

**Table 3:** Subjective sleep quality recorded pre- and during home confinement.

Parameters	Means ±SD		Δ (Δ%)	T (Wilcoxon)	Z	*p*-Value	ES
	Pre-Lockdown	During Lockdown					
Sleep latency (min)	19.99 ± 27.05	26.53 ± 39.18	6.54 (32.7%)	1042.5	8.56	<0.001	0.70
Sleep duration (h)	6.80 ± 1.23	6.96 ± 1.42	0.16 (2.4%)	9946	3.30	<0.001	0.22
Subjective sleep quality (A.U)	0.90 ± 0.66	1.05 ± 0.77	0.15 (16.6%)	1340	5.66	<0.001	0.53
Time in bed (h)	7.99 ± 1.46	8.31 ± 1.56	0.32 (4%)	16,096.5	6.98	<0.001	0.38
Sleep efficiency (%)	86.10 ± 13.1	84.70 ± 14.7	−1.36 (−1.6%)	27,022.5	2.61	0.009	0.14
Sleep disturbance (A.U)	1.41 ± 0.64	1.53 ± 0.69	0.13 (9.1%)	728	5.67	<0.001	0.58
Daytime dysfunction (A.U)	0.80 ± 0.99	1.17 ± 1.24	0.37 (46.6%)	3755	7.28	<0.001	0.52
Use of hypnotic medication (A.U)	0.38 ± 0.85	0.44 ± 0.94	0.06 (17%)	292.5	3.47	<0.001	0.49
Total score of PSQI (A.U)	4.88 ± 2.86	5.69 ± 3.37	0.81 (16.7%)	15011	8.00	<0.001	0.43

SD: Standard deviation; Δ%: % change from pre- to during confinement period; A.U: arbitrary unit; ES: effect size; PSQI: Pittsburgh Sleep Quality Index.

**Table 4:** Responses to the short form of the International Physical Activity Questionnaire recorded pre- and during lockdown.

Parameters		Means ±SD		Δ (Δ%)	T (Wilcoxon)	Z	*p*-Value	ES
		Pre-Lockdown	During Lockdown					
Vigorous intensity	Days/week	1.95 ± 2.05	1.61 ± 2.1	−0.34 (−17.4%)	7523	4.82	<0.001	0.33
	min/week	37.84 ± 52.58	29.73 ± 50.13	−8.12 (−21.4%)	2688	5.92	<0.001	0.48
	MET values	954 ± 1807	783 ± 1868	−171 (−17.9%)	8671	4.90	<0.001	0.32
Moderate intensity	Days/week	2.38 ± 2.11	1.86 ± 2.24	−0.52 (−22%)	8943.5	6.26	<0.001	0.39
	min/week	45.21 ± 50.77	35.3 ± 49.59	−9.91 (−21.9%)	3626.5	6.68	<0.001	0.49
	MET values	574 ± 853	457 ± 844	−116 (−20.3%)	10,910	5.82	<0.001	0.35
Walking	Days/week	3.91 ± 2.39	2.89 ± 2.63	−1.03 (−26.2%)	9449.5	9.22	<0.001	0.52
	min/week	44.48 ± 45.86	36.58 ± 38	−7.9 (−17.8%)	7960	5.03	<0.001	0.33
	MET values	673 ± 870	518 ± 792	−155 (−23.1%)	17,299	6.24	<0.001	0.34
All PA	Days/week	5.62 ± 2.11	4.34 ± 2.73	−1.28 (−22.7%)	3263	11.25	<0.001	0.70
	min/week	128 ± 108	102 ± 106	−26 (−20.3%)	9886	8.72	<0.001	0.50
	MET values	2201 ± 2604	1759 ± 2748	−443 (−20.1%)	23,207.5	7.77	<0.001	0.38
Sitting	hours/day	5.33 ± 3.03	6.78 ± 3.47	1.45 (27.2%)	3416.5	12.99	<0.001	0.74

SD: Standard deviation; Δ%: % change from pre- to during lockdown period; ES: effect size; MET: Metabolic equivalent of task (MET-min·week^−1^); PA: physical activity.

**Table 5:** Summary of regression predicting ∆ mental wellbeing from socio-demographic and health-related variables, ∆ all PA, ∆ sitting and ∆ total PSQI score.

Models	Predictor Variable	UC		SC	T	*p*-Value	R	SEE	Adjusted R^2^	F	*p*-Value
		b	SE	β							
Model 1	(Constant)	−2.307	1.972		−1.170	0.242		3.31	0.015	1.99	0.045
	Age	0.006	0.028	0.011	0.225	0.822	−0.030				
	Sex	−0.283	0.303	−0.043	−0.935	0.350	−0.062				
	Continent	0.018	0.138	0.006	0.127	0.899	0.018				
	Level of education	0.346	0.178	0.089	1.947	0.052	0.120				
	Marital status	−0.232	0.285	−0.036	−0.815	0.415	−0.050				
	Employment status	−0.086	0.080	−0.054	−1.075	0.283	−0.084				
	Health status	−0.371	0.275	−0.061	−1.347	0.179	−0.087				
	Δ house members	0.456	0.295	0.068	1.548	0.122	0.084				
Model 2	(Constant)	−2.169	1.985		−1.093	0.275		3.31	0.014	1.82	0.063
	Age	0.004	0.028	0.007	0.147	0.883	−0.030				
	Sex	−0.288	0.303	−0.043	−0.949	0.343	−0.062				
	Continent	0.030	0.140	0.010	0.216	0.829	0.018				
	Level of education	0.346	0.178	0.089	1.943	0.053	0.120				
	Marital status	−0.226	0.285	−0.035	−0.793	0.428	−0.050				
	Employment status	−0.084	0.080	−0.052	−1.045	0.296	−0.084				
	Health status	−0.355	0.277	−0.058	−1.282	0.200	−0.087				
	Δ house members	0.469	0.296	0.070	1.586	0.113	0.084				
	Δ sitting	−0.043	0.068	−0.028	−0.634	0.527	−0.028				
Model 3	(Constant)	−1.838	1.903		−0.965	0.335		3.17	0.094	6.35	<0.001
	Age	−0.001	0.027	−0.002	−0.044	0.965	−0.030				
	Sex	−0.227	0.291	−0.034	−0.781	0.435	−0.062				
	Continent	−0.097	0.135	−0.032	−0.716	0.474	0.018				
	Level of education	0.362	0.171	0.093	1.941	0.054	0.120				
	Marital status	−0.208	0.273	−0.032	−0.761	0.447	−0.050				
	Employment status	−0.092	0.077	−0.057	−1.195	0.233	−0.084				
	Health Status	−0.256	0.266	−0.042	−0.965	0.335	−0.087				
	Δ house members	0.340	0.284	0.051	1.196	0.232	0.084				
	Δ sitting	0.059	0.067	0.039	0.878	0.380	−0.028				
	Δ All PA (MET values)	0.0004	0.0001	0.295	7.195	0.000	0.290				
Model 4	(Constant)	−1.607	1.782		−0.902	0.367		2.967	0.206	13.2	<0.001
	Age	−0.007	0.025	−0.013	−0.279	0.780	−0.030				
	Sex	−0.167	0.272	−0.025	−0.614	0.540	−0.062				
	Continent	−0.104	0.127	−0.034	−0.818	0.414	0.018				
	Level of education	0.346	0.160	0.089	1.734	0.067	0.120				
	Marital status	−0.275	0.256	−0.043	−1.077	0.282	−0.050				
	Employment status	−0.050	0.072	−0.031	−0.690	0.491	−0.084				
	Health status	0.161	0.253	0.027	0.637	0.524	−0.087				
	Δ house members	0.269	0.266	0.040	1.012	0.312	0.084				
	Δ sitting	0.047	0.063	0.031	0.747	0.456	−0.028				
	Δ All PA (MET values)	0.0004	0.0001	0.293	7.183	0.000	0.290				
	Δ PSQI	−0.518	0.061	−0.343	−8.526	0.000	−0.354				
Model 5	(Constant)	−1.777	0.142		−12.535	0.000		2.975	0.202	66.41	<0.001
	Δ All PA (MET values)	0.0004	0.0001	0.284	7.210	0.000	0.290				
	Δ PSQI	−0.525	0.059	−0.348	−8.854	0.000	−0.354				

UC: unstandardized coefficients; SC: standardized coefficients; SEE: standard error of the estimate; PSQI: Pittsburgh Sleep Quality Index; PA: physical activity, ∆: change in total score from pre- to during lockdown; MET: Metabolic equivalent of task (MET-min·week^−1^); R: coefficient of correlation, R^2^: adjusted coefficient of determination.

## Data Availability

Data are available from the authors (K.T., or A.A.) upon reasonable request.

## Supplementary Materials

The following are available online at https://www.mdpi.com/article/10.3390/ijerph18084329/s1. File S1: Sample size; File S2: Data Privacy and Consent of Participation; File S3: Survey Questionnaires.
