# Supplementary material for: Sleep Quality and Physical Activity as Predictors of Mental Wellbeing Variance in Older Adults during COVID-19 Lockdown: ECLB COVID-19 International Online Survey
Source: Int J Environ Res Public Health. 2021 Apr 19;18(8):4329. doi: 10.3390/ijerph18084329 (PMC8073845; doi:10.3390/ijerph18084329)
Supplement: Supplementary file 1 [file ijerph-18-04329-s001.zip › ijerph-1154694-supplementary.pdf]

International Journal of
Environmental Research and Public Health

MDPI

Supplementary Materials:

Sleep Quality and Physical Activity as Predictors of Mental Wellbeing Variance in Older Adults during COVID-19 Lockdown: ECLB COVID-19 International Online Survey

Khaled Trabelsi [1,2,†], Achraf Ammar [3,4,*,†] and ECLB-COVID19 Consortium [1]

Supplementary file 1

Sample size

The study sample size was estimated using the following predictive equation [1]: $N = (Z_{\alpha/2})^2\ s^2/d^2$, where "N" is the number of needed participants (*i.e.*, old adults), "$Z_{\alpha/2}$" is the normal deviate for a two-tailed alternative hypothesis at a level of significance, "s" is the standard deviation (SD) and "d" is the accuracy of estimate or how close to the true mean. Given the novel nature of our study, the "s" was collected from a previous study including 1809 adults [18–34 years (31.5%), 35–49 years (39.8%), and >50 years (28.7%)] and aimed to describe how positive health behaviors (physical activity, sleep) and negative health behaviors (alcohol use, tobacco use, drug use) changed during March to mid-April 2020 [2]. Sleep quality was measured using the Pittsburgh Sleep Quality Index (PSQI) [3]. In the aforementioned study, the mean±SD of the total sample PSQI scores was 6.5±3.3. The consideration of a "$Z_{\alpha/2}$" equal to 1.96 (*i.e.*, an error rate of 0.05%) and an arbitrarily "d" equal to an error of estimation less than 0.32 (5% of the mean of 6.5), to provide an estimated sample size of $N = (1.96)^2\ (3.3)^2/(0.32)^2 = 409$ participants. The assumption of 20% for missing data, data entry errors, or the occurrence of a cognitive decline/impairment gave a revised sample of 511 old adults [511 = 409/ (1.00-0.20)].

Supplementary file 2

Data Privacy and Consent of Participation

The ECLB-COVID-19 study gave special care to data privacy and security and protection of the collected data against any unauthorized access by third parties, During the informed consent process, surveyed participants were ensured ***(i)*** all data would be used only for research purposes ***(ii)*** answers were anonymous and confidential according to Google's privacy policy. Participants were not permitted to provide their names or contact information. Additionally, participants were able to stop study participation and leave the questionnaire at any stage before the submission process; if doing so, their responses would not be saved. Responses were saved only by clicking on the provided "submit" button. Participants were requested to be honest in their responses **[4]**.

Survey Questionnaires

The ECLB-COVID-19 is a multi-country electronic survey designed to assess eventual changes in multiple lifestyle behaviours during the COVID-19 outbreak. Therefore, a collection of validated and/or crisis oriented brief questionnaires were included. These questionnaires assessed demographic information, mental wellbeing (Short Warwick-Edinburgh Mental Wellbeing Scale (SWEMWBS) **[5]**, mood and feeling (Short Mood and Feelings Questionnaire (SMFQ), life satisfaction (Short Life Satisfaction Questionnaire for Lockdowns (SLSQL) **[6]**, social participation (Short Social Participation Questionnaire for Lockdowns (SSPQL) **[6]**, PA (International Physical Activity Questionnaire Short Form (IPAQ-SF) **[4]**, diet behaviours (Short Diet Behaviours Questionnaire for Lockdowns (SDBQL) **[4]**, sleep quality (Pittsburgh Sleep Quality Index (PSQI) and some key questions assessing the technology-use behaviours (Short Technology-use Behaviours Questionnaire for Lockdowns (STBQL)), and the need of psychosocial support. Reliability of the shortened and/or newly adopted questionnaires was tested by the project steering group through piloting, prior to survey administration. These brief crisis-oriented questionnaires demonstrated good to excellent

test–retest reliability coefficients (r = 0.84–0.96). A multi-language validated version already existed for the majority of these questionnaires and/or questions. However, for questionnaires that did not already exist in multilingual versions, we followed the procedure of translation and back-translation, with an additional review for all language versions from the international scientists of our consortium. As a result, a total number of 64 items were included in the ECLB-COVID-19 online survey in a differential format (***i.e.***, each item or question requested two answers, one regarding the period pre- and the other regarding the period during- lockdown). The participants were guided to compare the situations. A copy of the complete ECLB-COVID19 survey's questionnaires has been previously published as supplementary file (**https://doi.org/10.1371/journal.pone.0240204.s001**).

Supplementary file 3

SWEMWBS

SWEMWBS is a short version of the Warwick–Edinburgh Mental Wellbeing Scale (WEMWBS) **[5]**. The SWEMWBS uses seven of the WEMWBS's 14 statements about thoughts and feelings, which relate more to functioning than feelings suggesting an ability to detect clinically meaningful change **[8]**. The seven statements are positively worded with five response categories from 'none of the time (score 1)' to 'all of the time (score 5)'. The SWEMWBS was recently validated for the general population and is scored by first summing the scores for each of the seven items, which are scored from 1 to 5 **[9]**. The total raw scores are then transformed into metric scores using the SWEMWBS conversion table **[9]**. Total scores range from 7 to 35 with higher scores indicating higher positive mental wellbeing **[9]**. The cut points for SWEMWBS are 17 or less for probable depression, 18-20 for possible depression, 21-27 for average mental wellbeing and 28-35 high mental wellbeing **[9]**.

PSQI

The sleep quality was assessed by the PSQI **[10]**. The PSQI questionnaire is reliable and valid assessment tool in older adults **[11]**. The questionnaire was composed of 19 questions representing one of the seven components of sleep quality: subjective sleep quality, sleep latency, sleep duration, sleep efficiency, sleep disturbance, sleep medication intake, and daytime dysfunction. Each component score was rated on a 3-point scale, leading to a sum of up to 21 points. PSQI scores > 5 and ≤ 5 indicated, respectively, poor and good sleep qualities.

IPAQ-SF

According to the official IPAQ-SF guidelines **[7]**, data from the IPAQ-SF are summed within each item (**i.e.**, vigorous intensity, moderate intensity and walking) to estimate the total amount of time spent engaged in PA per week. Total weekly PA (MET-min·week^{-1}) was estimated by adding the products of reported time for each item by a Metabolic equivalent of task (MET) value that was specific to each category of PA. We assigned two different sets of MET values. The first set was the original values (original IPAQ) based on the official IPAQ guidelines for young and middle-aged adult (18–65 years old): vigorous PA = 8.0 METs, moderate PA = 4.0 METs and walking = 3.3 METs. The other set used modified values (modified IPAQ), which we had devised for use with older adults (>65 years old), as reported by Stewart et al**.** **[12]** and Yasunaga et al. **[13]**: vigorous PA = 5.3 METs, moderate PA = 3.0 METs and walking = 2.5 METs. Additionally, we added the total PA (sum of performed vigorous, moderate and walking activity) as a fourth item and sitting time as fifth item **[4,14,15]**.

Based on the IPAQ recommendations for scoring protocol, participants of the study were classified in three different groups of PA considering the MET–min/wk of the sum of walking, moderate-intensity physical activities, and vigorous-intensity physical activities: lowly active (<600 MET–min/wk); moderately active (600 MET–min/wk ≤ PA < 3000 MET–min/wk) and highly active (≥3000 MET–min/wk) (**http://www.ipaq.ki.se**).

References for supplementary files

1. Serhier, Z.; Bendahhou, K.; Ben Abdelaziz, A.; Bennani, M.O. Methodological sheet n°1: How to calculate the size of a sample for an observational study? Tunis Med 2020 Jan, 98(1), 1-7. PMID: 32395771
2. Knell, G.; Robertson, M.C.; Dooley, E.E.; Burford, K.; Mendez, K.S. Health Behavior Changes During COVID-19 Pandemic and Subsequent "Stay-at-Home" Orders. Int. J. Environ. Res. Public Health 2020, 17, 6268.

3. Buysse, D.J.; Reynolds, C.F.; Monk, T.H.; Berman, S.R.; Kupfer, D.J. The Pittsburgh Sleep Quality Index: A new instrument for psychiatric practice and research. Psychiatry Res. 1989, 28, 193–213.
4. Ammar, A.; Brach, M.; Trabelsi, K.; Chtourou, H.; Boukhris, O.; Masmoudi, L.; Bouaziz, B.; Bentlage, E.; How, D.; Ahmed, M.; et al. on behalf of the ECLB-COVID19 Consortium. Effects of COVID-19 Home Confinement on Eating Behaviour and Physical Activity: Results of the ECLB-COVID19 International Online Survey. Nutrients 2020, 12, 1583. DOI: 10.3390/nu12061583
5. Stewart-Brown, S.; Tennant, A.; Tennant, R.; Platt, S.; Parkinson, J.; Weich, S. Internal construct validity of the Warwick-Edinburgh Mental Wellbeing Scale (WEMWBS): A Rasch analysis using data from the Scottish Health Education Population Survey. Health Qual. Life Outcomes 2009, 7(1), 1–8. doi:10.1186/1477-7525-7-15.
6. Ammar, A.; Chtourou, H.; Boukhris, O.; Trabelsi, K.; Masmoudi, L.; Brach, M.; Bouaziz, B.; Bentlage, E.; How, D.; Ahmed, M.; et al. on behalf of the ECLB-COVID19 Consortium. COVID-19 home confinement negatively impacts social participation and life satisfaction: a worldwide multicentre study. Int J Environ Res Public Health 2020, 17(17), 6237; DOI:10.3390/ijerph17176237.
7. IPAQ Research Committee, International Physical Activity Questionnaire: Short Last 7 Days Self-Administered Format, 2005. http//www.Ipaq.Ki.Se.
8. Fat, L.N.; Scholes, S.; Boniface, S.; Mindell, J.; Stewart-Brown, S. Evaluating and establishing national norms for mental wellbeing using the short Warwick–Edinburgh mental wellbeing scale (SWEMWBS): findings from the health survey for England. Qual Life Res 2017, 26 (5), 1129–1144. https://doi.org/10.1007/s11136-016-1454-8.
9. Warwick Medical School. (2020). Warwick medical School. *Collect, score, analyse and interpret WEMWBS*. https://warwick.ac.uk/fac/sci/med/research/platform/wemw
10. Buysse, D.J.; Reynolds, C.F.; Monk, T.H.; Berman, S.R.; Kupfer, D.J. The Pittsburgh Sleep Quality Index: A new instrument for psychiatric practice and research. Psychiatry Res. 1989, 28, 193–213.
11. Chehri, A.; Nourozi, M.; Eskandari, S.; Khazaie, H.; Hemati, N.; Jalali, A. Validation of the Persian version of the Pittsburgh Sleep Quality Index in elderly population. *Sleep Sci* 2020, *13*(2), 119.
12. Stewart AL, Mills KM, King AC, Haskell WL, Gillis DAWN, Ritter PL. CHAMPS physical activity questionnaire for older adults: Outcomes for interventions. Med. Sci. Sports Exerc. 2001; 33: 1126–1141.
13. Yasunaga A, Park H, Watanabe E, Togo F, Park S, Shephard RJ, Aoyagi Y. Development and evaluation of the physical activity questionnaire for elderly Japanese: The Nakanojo study. J. Aging Phys. Act. 2007; 15: 398–411.
14. Ammar, A.; Trabelsi, K.; Brach, M.; Chtourou, H.; Boukhris, O.; Masmoudi, L.; Bouaziz, B.; Bentlage, E.; How, D.; Ahmed, M.; et al. on behalf of the ECLB-COVID19 Consortium. Effects of home confinement on mental health and lifestyle behaviours during the COVID-19 outbreak: Insight from the ECLB-COVID19 multicenter study. Biol Sport 2020. DOI:10.5114/biolsport.2020.96857.
15. Trabelsi, K.; Ammar, A.; Masmoudi, L.; Boukhris, O.; Chtourou, H.; Bouaziz, B.; Brach, M.; Bentlage, E.; How, D.; Ahmed, M.; et al. Globally altered sleep patterns and physical activity levels by confinement in 5056 individuals: ECLB COVID-19 international online survey. *Biol Sport* 2021, *38*(4), 495-506.
